# Seasonal and Temporal Ensemble Models for Accurate Near-Surface Air Temperature Estimation

**DOI:** 10.3390/s24237507

**Published:** 2024-11-25

**Authors:** Rey Jalbuena, Jurng-Jae Yee

**Affiliations:** Department of ICT Integrated Ocean Smart Cities Engineering, Dong-A University, Busan 49315, Republic of Korea; 2076946@donga.ac.kr

**Keywords:** MODIS LST, near-surface air temperature, regression modeling, spatial analysis, thermal environment

## Abstract

The near-surface air temperature (NSAT) is crucial for understanding thermal and urban environments. Traditional estimation methods using general remote sensing images often focus on the types of spatial data or machine learning models used, neglecting the importance of seasonal and temporal variations, limiting their accuracy. This study introduces a novel ensemble model that incorporates both seasonal and temporal information integrated with satellite-derived land surface temperature (LST) data to enhance NSAT estimation, along with a rigorous feature importance analysis to identify the most impactful parameters. Data from 2022, collected from 147 South Korean weather stations, were used to develop and evaluate the models. Thirteen initial variables, including the LST and other auxiliary data, were considered. Random forest regression was employed to build separate models for each season. This novel approach of separating data by season allowed optimized feature selection tailored to each season, improving the model efficiency and capturing finer seasonal and daily temperature variations. These seasonal models were then combined to form an ensemble model. The seasonal models demonstrated varying accuracy, with the R^2^ values indicating a strong correlation between the predicted and actual NSAT, particularly high in spring and fall and lower in summer and winter. The ensemble model showed improved performance, achieving an MAE of 0.534, an RMSE of 0.391, an R^2^ of 0.996, and a cross-validated R^2^ of 0.968. These findings highlight the effectiveness of incorporating seasonal and temporal information into NSAT estimation models, offering significant improvements over traditional approaches. The developed models support precise temperature monitoring and forecasting, aiding environmental and urban management.

## 1. Introduction

The near-surface air temperature (NSAT) refers to the air temperature of just about the Earth’s surface, typically measured at 1.5 to 2 m above ground level [1]. The NSAT is a pivotal parameter in environmental monitoring, climate studies, and urban planning due to its influence on various ecological and meteorological processes. Accurate NSAT data are essential for understanding the Earth’s climate system dynamics, including weather patterns, seasonal changes, and long-term climate trends. It affects evapotranspiration, soil moisture dynamics, and vegetation growth and distribution [2]. Monitoring the NSAT allows scientists to track the effects of climate change, such as global warming, on regional and local scales, providing valuable insights into the changing climate and its impacts on natural and human systems [3]. In urban planning, NSAT data are crucial for managing and mitigating the urban heat island (UHI) effect, where urban areas experience higher temperatures than their rural surroundings due to human activities and altered land surfaces [4]. Accurate NSAT information helps city planners and policymakers design more sustainable and resilient cities by implementing strategies such as increasing green spaces, improving building designs, and optimizing urban layouts to reduce heat retention [5,6]. Additionally, NSAT data support public health initiatives by forecasting heatwaves and other extreme weather events, enabling timely interventions to protect vulnerable populations [7].

The most common source of NSAT data is ground-based meteorological stations. While valuable for weather and climate monitoring, these stations face significant limitations due to their sparse distribution, particularly in remote and rural areas, leading to data gaps that hinder accurate regional and global climate analysis [8]. The maintenance and operation of these stations are resource-intensive, requiring regular calibration and upkeep to ensure data accuracy and reliability [9]. To address these limitations, satellite-derived data, such as the land surface temperature (LST), have emerged as a valuable alternative for large-scale NSAT estimation. One source, Moderate Resolution Imaging Spectroradiometer (MODIS) LST data, offers extensive spatial coverage and frequent temporal resolution, providing global observations at a spatial resolution of 1 km and a daily temporal resolution, which allows for continuous monitoring of temperature dynamics across diverse landscapes [10]. These high-resolution data fill the gaps left by the sparse distribution of ground-based stations, enabling a more detailed analysis of temperature patterns and trends. For instance, the integration of satellite data with ground-based observations has been shown to improve the accuracy of agrometeorological services, enhancing decision-making in agriculture by providing precise meteorological conditions and vegetation states [11].

Additionally, the use of reanalysis datasets, which combine satellite and ground-based data, has proven effective in epidemiological studies, offering consistent spatiotemporal coverage and reliable temperature-related health risk assessments [12]. Furthermore, ground-based multi-channel microwave radiometers (MWRs) and other advanced ground-based sensors can supplement satellite observations, improving numerical weather prediction and real-time monitoring [13]. Overall, leveraging MODIS LST data and integrating it with ground-based observations and reanalysis datasets can significantly enhance our understanding of climate processes, improve climate monitoring, and support urban planning initiatives, thereby addressing the limitations of relying solely on ground-based meteorological stations [14,15].

This is why precise and accurate MODIS LST-derived NSAT data are crucial. Various methods, including statistical downscaling, machine learning models, and remote sensing data integration, have been developed to enhance the accuracy of NSAT estimations from MODIS LST to ensure data integrity [16,17]. LST-based NSAT estimation has been a focus of numerous studies, each employing different methodologies to enhance the accuracy. For instance, a study in Georgia, USA, utilized linear regression, support vector regression (SVR), and hybrid artificial neural networks, achieving an accuracy of 2.14 °C with a correlation coefficient (R) of 0.96 using the hybrid model [18]. In the Jingjinji area of China, the RF algorithm was found to be the most effective, yielding a daily RMSE of 1.29 °C, an MAE of 0.94 °C, and a daily average R^2^ of 0.99. The daytime and nighttime instantaneous RMSEs were 1.88 °C and 2.47 °C, respectively, with corresponding MAEs of 1.35 °C and 1.83 °C [19]. Another study developed a fully coupled framework integrating physical, statistical, and deep learning (DL) methods (PS-DL), achieving an RMSE of 0.89 K and an MAE of 0.78 K in the best-case scenario, with cross-validation against the China Meteorological Forcing Dataset showing an RMSE of 1.29 K and an MAE of 1.00 K [20]. For the Tibetan Plateau, a novel model using machine learning techniques and all-weather LST data achieved an overall RMSE of 2.43 °C and an R^2^ of 0.93, demonstrating reliable performance even in cloudy conditions [21]. In the Republic of Korea, a deep neural network (DNN) model using various input variables, including NDVI and 11 and 12 µm band data, achieved a correlation coefficient of 0.98 and an RMSE of 2.19 K, with seasonal analysis showing the lowest accuracy in spring (RMSE of 2.82 K) and higher accuracy in other seasons (RMSE under 2 K) [22]. These studies collectively highlight the significant role of the LST in accurately estimating the NSAT, with varying degrees of success depending on the methodology and regional characteristics.

Although the estimation of the NSAT using LST data has proven advantageous for large-scale temperature monitoring, it still faces significant challenges due to seasonal variability. Seasonal changes impact the LST–NSAT relationship through variations in vegetation cover, snow presence, and solar radiation. For instance, snow cover reflects solar radiation during winter, leading to lower LSTs than actual air temperatures, creating discrepancies if the albedo effect is not accounted for [23]. Similarly, MODIS LST data tend to overestimate the temperature variability in summer and underestimate it in winter, with biases of up to 6.7 °C in summer and minimal biases in winter [24]. Increased solar radiation and reduced vegetation cover in summer can result in higher LSTs, which may not accurately reflect the NSAT, especially in densely vegetated areas that cool more rapidly than the surface [25]. The correlation between the LST and the NSAT is stronger in winter than in summer, particularly in sparsely vegetated and urban areas, as observed in several studies [26]. Traditional models often struggle with these variations as they treat data uniformly across different seasons, failing to capture the unique environmental conditions influencing temperature relationships in each period [27]. During spring and fall, transitional periods with rapidly changing vegetation and temperature patterns, the LST–NSAT relationship can be highly dynamic and challenging to model accurately without considering these specific seasonal effects [28]. The relationship between the LST and vegetation indices like the NDVI also varies seasonally, being more stable in winter than in summer [29]. Given these seasonal complexities, developing separate models tailored to each season could potentially enhance the accuracy of NSAT estimation by better aligning with seasonal environmental variations. However, to date, no study has attempted to develop separate models for each season to fully capture these seasonal dynamics. Integrating seasonal variations into machine learning models can improve the accuracy of NSAT estimations derived from MODIS LST data. Furthermore, machine learning models, such as random forest (RF) and hybrid artificial neural networks, have shown promise in enhancing the predictive performance of NDVI reconstruction and LST modeling by incorporating time and location variables, significantly improving the model accuracy across different seasons [30,31]. Therefore, adapting models to seasonal dynamics is essential for achieving dependable year-round NSAT estimates from MODIS LST data.

This study proposes developing temporally and seasonally informed machine learning models to address the challenges of seasonal variability in NSAT estimation. Unlike traditional models that treat data uniformly across seasons, our approach integrates specific seasonal features into the machine learning framework. This study presents several key novelties: (1) the use of temporal data, such as the day and month, to achieve more detailed, intra-daily NSAT estimates and capture finer seasonal and daily temperature variations; (2) a rigorous feature importance analysis to identify and prioritize the most impactful variables, eliminating less significant parameters to streamline the model and enhance the predictive accuracy; and (3) the development of a seasonal ensemble model, where separate models are tailored to the unique environmental conditions of each season. By incorporating variables such as vegetation indices, cloud cover, land surface albedo, and other seasonally relevant data, the models can better account for the dynamic environmental conditions influencing the LST–NSAT relationship throughout the year. This method aims to enhance the predictive accuracy of NSAT estimates by tailoring the model’s learning process to recognize and adapt to seasonal patterns and transitions. Methods such as machine learning and ensemble models have shown promise in enhancing predictive performance by incorporating time and location variables, significantly improving the model accuracy across different seasons. Therefore, adapting models to seasonal dynamics is essential for achieving dependable year-round NSAT estimates from MODIS LST data.

The primary research questions guiding this study are as follows. What are the key spatio-temporal factors influencing the LST–NSAT relationship during different seasons? How can these factors be integrated into machine learning models to improve the estimation accuracy? What is the best method to handle these seasonal data? To address these questions, the specific objectives of this research are to identify and analyze key seasonal environmental factors, integrate these factors into machine learning models, and evaluate the model performance across seasons.

## 2. Materials and Methods

### 2.1. Study Area

**Figure 1:**
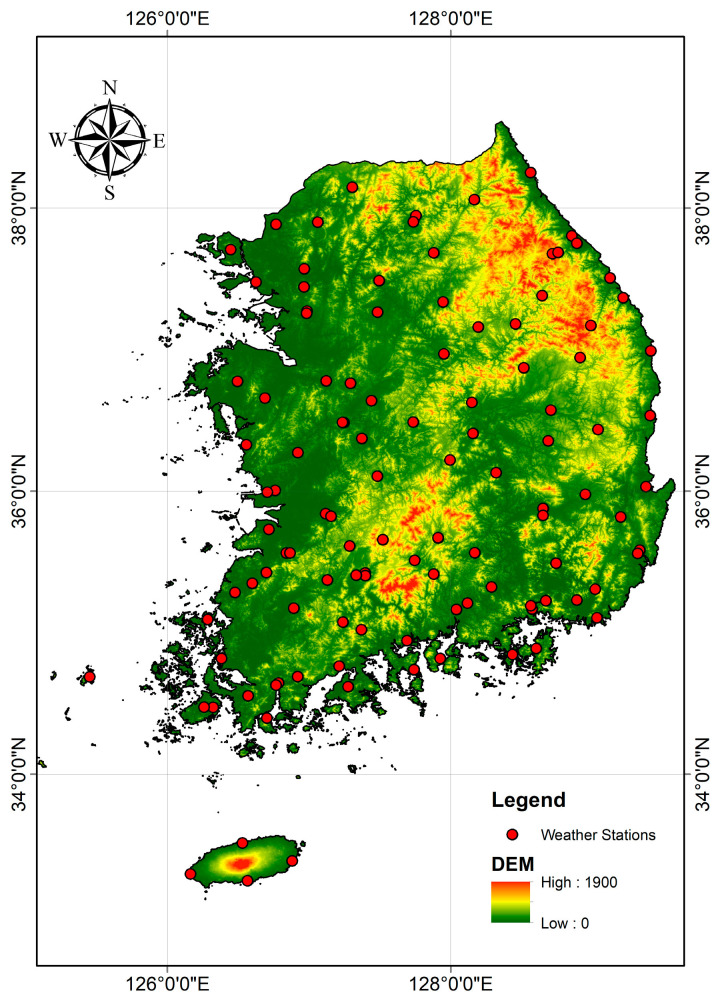
Location of the ground stations located in Korea with a DEM basemap in meters.

The study area chosen for this research is the Republic of Korea, a region characterized by diverse climatic conditions and landscape features. The Republic of Korea experiences distinct seasonal variations, with cold winters marked by significant snow cover and hot, humid summers [32]. According to the Korea Meteorological Administration (KMA), the Republic of Korea’s climate is divided into four seasons based on traditional seasonal months: spring (March to May), summer (June to August), fall (September to November), and winter (December to February) [33]. Spring gradually brings warm temperatures from around 5 °C to 20 °C, with blooming flowers like cherry blossoms and increasing humidity. Summer is hot and humid, with temperatures ranging from 25 °C to over 35 °C and heavy rainfall due to the monsoon season, leading to lush vegetation. Fall offers mild, dry weather, with temperatures dropping from 20 °C to 5 °C, and vibrant autumn foliage. Winter is cold and dry, with temperatures ranging from −5 °C to 5 °C, accompanied by significant snowfall in northern and mountainous regions [34,35,36]. Figure 1 depicts the study area of South Korea, showing elevation through a DEM gradient and the distribution of weather stations.

These seasonal changes significantly impact the LST–NSAT relationship through variations in vegetation cover, snow presence, and solar radiation. During winter, regions with extensive snow cover, like Gangwon-do, can experience lower LSTs than actual air temperatures due to the reflection of solar radiation, potentially causing discrepancies if the albedo effect is not considered. Snow cover significantly influences the surface albedo, with studies emphasizing the importance of accurately simulating the snow cover distribution to improve winter climate simulations [37]. Conversely, increased solar radiation and reduced vegetation cover during the summer can result in higher LSTs, which may not accurately reflect the NSAT, particularly in densely vegetated areas like the southern coastal regions and Jeju Island. Studies have shown a significant increase in the LST over time, with a stronger rise during summertime, indicating a spatial coherence between LST changes and vegetation coverage alterations [38]. Additionally, urban areas like Seoul and Busan exhibit unique thermal properties. Studies show that the correlation between the LST and the NSAT is stronger in winter than in summer due to the urban heat island effect and heterogeneous surfaces [39,40]. Integrating seasonal variations into machine learning models is crucial to improving the accuracy of NSAT estimations in the Republic of Korea. With these considerations in mind, the researchers believe that the Republic of Korea provides an ideal setting for this research, offering a comprehensive environment in which to study the impacts of seasonal variability on LST–NSAT relationships.

### 2.2. Data

This study used hourly NSAT data from 164 KMA stations for 2022, aligned with MODIS Terra and Aqua satellite daytime observations, to estimate the NSAT from the LST [41]. However, after inspection, 17 stations were found to be decommissioned and lacking data for 2022, reducing the final dataset to 147 stations. For satellite data, this study utilized MODIS daily LST products (MOD11A1 and MYD11A1), covering all 365 days of 2022. MODIS was selected for its daily observation capability, high temporal resolution, and broad spatial coverage [29], aligning well with this study’s objectives.

**Figure 2:**
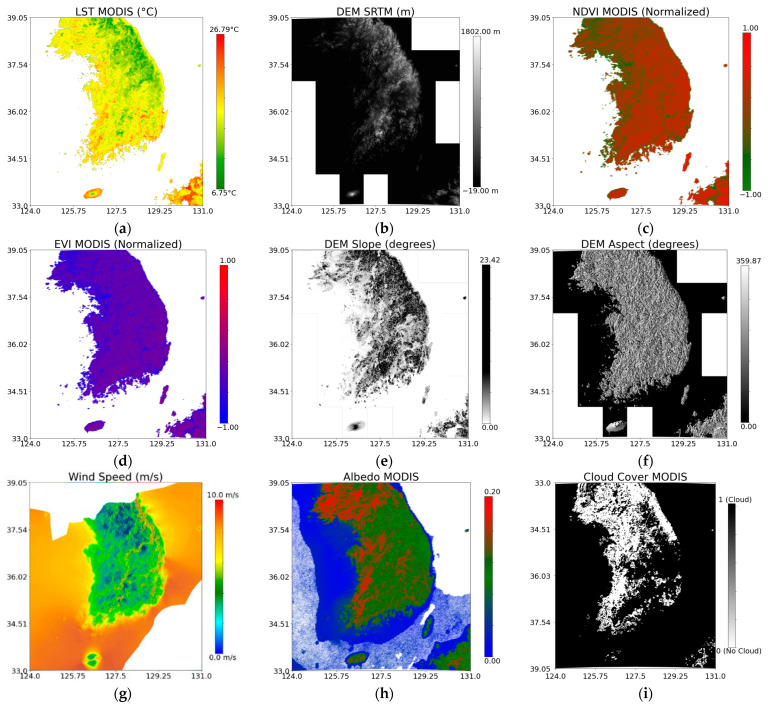
The 2022 mean annual value images of the dataset used: (**a**) LST, (**b**) DEM, (**c**) NDVI, (**d**) EVI, (**e**) slope, (**f**) aspect, (**g**) mean wind speed, (**h**) surface albedo, and (**i**) cloud cover.

To aid in the NSAT estimation, incorporation of additional variables such as the digital elevation model (DEM), slope, aspect, normalized difference vegetation index (NDVI), enhanced vegetation index (EVI), surface albedo, cloud cover, and mean wind speed was performed. The surface albedo, derived from the MODIS MCD43A3 product using the white-sky albedo band, is crucial for understanding solar energy absorption at the surface, which significantly impacts the temperature distribution [42]. DEM data from the Shuttle Radar Topography Mission (SRTM) providing high-resolution topographic information for accounting for altitude-related temperature variations [43] was used. Then, the slope and aspect were derived from said DEM data. The NDVI and EVI, indicating vegetation health and density, were sourced from the MODIS MOD13A1 16-day composite product and were key factors in the air temperature modeling due to their influence on surface heat fluxes [44]. The mean wind speed data from the Global Wind Atlas (GWA) measured at 10 m above ground level were included to account for wind-induced heat and moisture transfer, aligning with methodologies used in previous studies to ensure consistency [45]. Lastly, cloud cover was derived from the QC band of the MODIS LST. The integration of these variables has been shown to significantly enhance the accuracy of NSAT estimations across various settings by addressing critical elements such as solar reflection, topography, vegetation health, and wind effects [46,47]. Summarized in Table 1 are the datasets utilized in this study, detailing the resolution and data source, while Figure 2 shows the images used.

All the images were resampled to 1 km to ensure the homogeneity of the analysis. Additionally, the location (latitude and longitude) and time (day and month) were used as additional X-variables for the prediction modeling, giving a total of 13 X-variables.

### 2.3. Methodology

#### 2.3.1. Data Preparation

**Figure 3:**
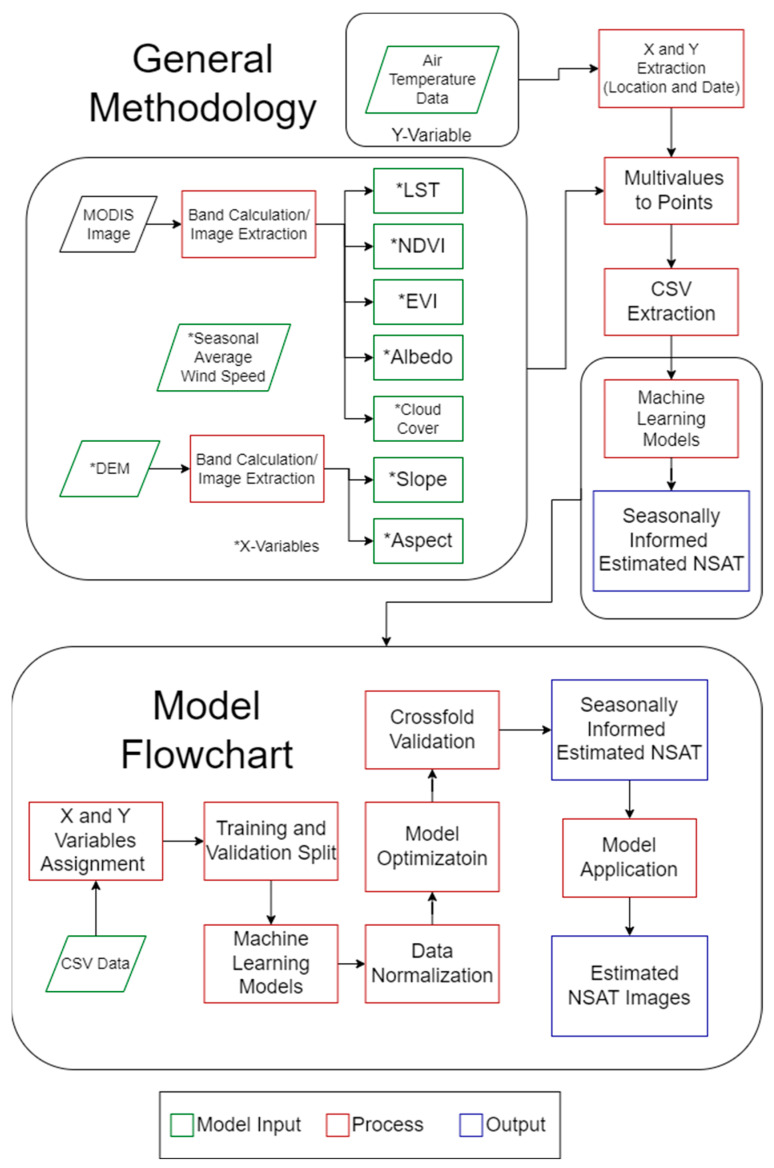
The general methodology of this study from data preparation to modeling, and lastly, results analysis.

As seen in Figure 3, the methodology was divided into data preparation and machine learning modeling. We extracted all the data from the images to coincide with the data points of the meteorological stations during the data preparation. Each meteorological data point was assigned a unique value for the LST and NSAT daily, while the albedo and NDVI values varied every 16 days. The remaining variables, like the DEM, remained constant for each point. Lastly, the average wind speed was for the four seasons. These data extracted from the images served as the X-variables, while the NSAT data were the Y-variable. Additionally, the location of the points and the date of the data were also used as X-variables. The LST and NSAT data were expressed in degrees Celsius. The data were then fed to the machine learning methods chosen.

The modeling flowchart in Figure 3 shows the data were partitioned into training and testing sets to facilitate the development and subsequent evaluation of the predictive models. The data were split with a 70% training and 30% testing ratio. Before the model training, we normalized the features to ensure a standardized scale. With the data suitably prepared, the models were constructed and trained. Each model underwent optimization, which involved tuning the hyperparameters. Model optimization seeks to find the best set of hyperparameters that lead to the highest prediction accuracy and generalization capability.

#### 2.3.2. Modeling Approaches

We explore and compare different distinct modeling approaches to improve the NSAT estimation from environmental variables. Each method leverages different strategies to capture the complexities of the dataset, aiming to identify the most effective technique for accurate temperature prediction.

The first approach involves developing a single machine learning model trained on the entire dataset to predict the air temperature. All the environmental variables are combined into a single dataset, and an RF model is trained on these comprehensive data. RF is an ensemble learning method used for classification and regression tasks. It builds multiple decision trees during training and outputs the mode of the classes or mean prediction of the individual trees [48]. It utilizes a combination of random subsets of training data and features in each tree to promote diversity, preventing overfitting and enhancing the generalization capabilities. Averaging the outcomes from multiple trees increases the accuracy and robustness. Additionally, we will compare the model’s performance with (RFWT) and without date (RFWOT) features to evaluate the impact of temporal variables on the prediction accuracy. This approach provides a baseline for comparison against more complex models and is straightforward to implement and manage.

The next approach involves developing distinct models for each season to account for seasonal variations in the data. The dataset is divided into four subsets based on the seasons (winter, spring, summer, and fall). Separate RF models are trained for each season. This novel approach of using separate models for each season allows the framework to capture unique seasonal characteristics more accurately, enhancing the prediction performance across variable environmental conditions. Then, the models are combined into one ensemble model at the end. This approach leverages the strengths of multiple specialized models through an ensemble technique. This approach aims to improve the overall prediction accuracy by mitigating individual model weaknesses and leveraging their strengths. There are again two distinct models for this approach, the data with temporal data (SEWT) and the model without the temporal data (SEWOT).

The last approach involves feature selection within the data parameters. To identify the optimal combination of features for each model, we explore all the combinations of the given parameters. Specifically, we evaluate the models using combinations ranging from two features up to the full set of thirteen features. This exhaustive search includes calculating all the combinations, resulting in a total of 8178 combination, to obtain the best set of data that produces the highest accuracy. This comprehensive approach ensures that we identify the feature sets that most significantly impact the model’s performance, leading to the selection of the best possible combination for accurate NSAT estimation. Unlike most studies that rely on predefined feature sets or those recommended in the previous literature, we take the time to assess the importance of each feature systematically, selecting only the variables that contribute most significantly to improving the accuracy. The feature importance and feature selection are gathered during this process by assessing how each feature contributes to improving the model accuracy. Using the RF algorithm, each feature’s impact is measured by evaluating its ability to reduce uncertainty across the ensemble of decision trees. This provides an importance value for each feature, indicating its predictive value and highlighting which variables have the most significant impact on the accuracy across seasons. There are two distinct models for this final approach, the first is the ensemble model with optimized parameters for the whole dataset (SEOP), and the second is the ensemble model with the best set of parameters distinct per season (SEOPPS). Overall, six models are developed, compared, and evaluated to determine not only which model produced the best results but also to assess whether the increased accuracy of more complex models justifies the additional resources required. Table 2 shows a summary of the six models used in this study.

To further enhance the performance of the models, hyperparameter tuning is conducted using grid search and cross-validation techniques. Hyperparameters such as the number of trees in the RF (50, 100, 200), maximum depth (none, 10, 20), minimum sample split (2, 5, 10), and minimum leaf samples (1, 2, 4) are implemented to identify the optimal settings for each model. This process involves evaluating the model performance across a range of hyperparameter values and selecting the combination that yields the best performance metrics.

In our study, we employ K-fold cross-validation (CV) to robustly evaluate the performance and generalizability of our machine learning models. K-fold CV is a statistical method used to estimate the skill of a model on new data, ensuring that the model is not overly tuned to the training data and can generalize well to unseen data. Specifically, we divide our dataset into *K* equally sized folds. The model is trained on *K* – 1 folds for each fold and evaluated on the remaining fold. This process is repeated *K* times, with each fold serving as the test set once. The results are then averaged to produce a single performance metric, calculated as follows:

(1) $$Average\;Perofrmance=\frac{1}{k}\sum_{i=1}^{K}Performance\;({Fold}_{i})$$

where performance (Fold *i*) represents the performance metric (such as accuracy, precision, recall, or RMSE) obtained from the *i*-th fold. This approach offers several advantages. By averaging the performance across multiple folds, we mitigate the impact of any single partitioning of the data, leading to a more reliable and robust assessment of model performance. Additionally, K-fold cross-validation allows us to use our entire dataset efficiently, as each data point is used for training and validation. In our experiments, we set *K* to 5, ensuring a balance between the computational efficiency and the reliability of the performance estimates. This methodology provides a comprehensive evaluation of our models, highlighting their strengths and potential areas for improvement.

This methodology is implemented using Python, with the key libraries including pandas, numpy, sklearn, matplotlib, seaborn, geopandas, and folium. These tools facilitate data processing, model training, and visualization, ensuring a robust and comprehensive analysis. By comparing these methods, this study aims to identify the most effective modeling strategy, considering factors like the seasonal variability and ensemble advantages, to achieve accurate and reliable air temperature predictions.

#### 2.3.3. Accuracy Assessment

For the accuracy assessment of the models, this research used three indicators, the root mean squared error (RMSE), the mean absolute error (MAE), and the coefficient of determination (R^2^), to verify the estimation results. The RMSE measures the magnitude between the predicted and actual values. The MAE refers to the absolute difference between the predicted and actual values. The R^2^ indicates the proportion of variance in the actual values explained by the model. Better model predictions will have smaller RMSE and MAE values and higher R^2^ values. The three metrics were calculated as follows.

(2) $$\mathrm{RMSE}=\sqrt{\frac{1}{n}\sum_{i=1}^{n}{\left({NSAT}_{R}-{NSAT}_{P}\right)}^{2}}$$

(3) $$\mathrm{MAE}=\frac{1}{n}\sum_{i=1}^{n}\left|{NSAT}_{R}-{NSAT}_{P}\right|$$

(4) $$R^{2}=1-\frac{\sum_{i=1}^{n}{\left({NSAT}_{R}-{NSAT}_{P}\right)}^{2}}{\sum_{i=1}^{n}{\left({NSAT}_{R}-\bar{{NSAT}_{P}}\right)}^{2}}$$

(5) $$CVR^{2}=\frac{1}{k}\sum_{i=1}^{k}R_{i}^{2}$$

where NSAT_P_ and NSAT_R_ denote the predicted NSAT value and the NSAT reference value, respectively. Furthermore, aside from R^2^ values, this study also presented the CV R^2^, which is the average R^2^ score obtained from the cross-validation, providing a robust measure of the model’s predictive performance and generalizability.

#### 2.3.4. Test Bed Area

**Figure 4:**
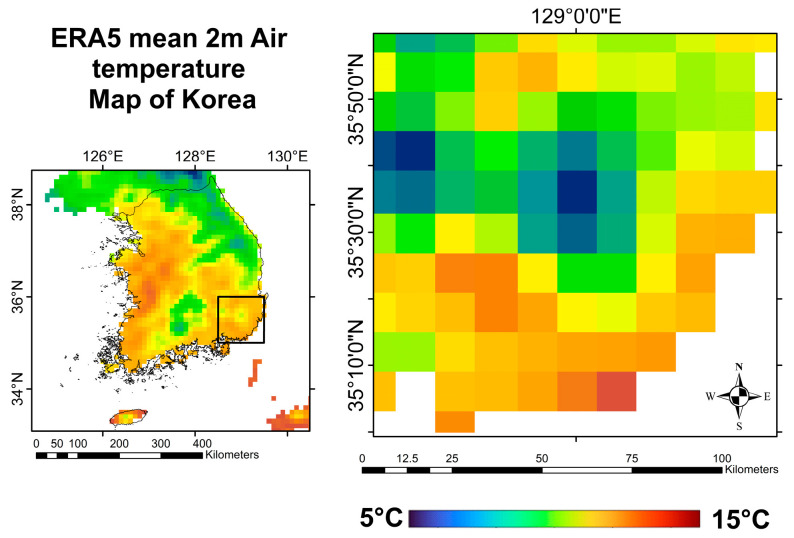
NSAT prediction test area. A 10 km × 10 km area in the middle of Busan, the Republic of Korea.

To have a benchmark for the accuracy of the NSAT models, this study used a test bed area covering a 100 km × 100 km region that includes Busan, Gyeongsangnam-do, and Ulsan, encompassing diverse land cover types, such as urban, mountainous, and coastal zones. The predictions from each model were compared against the ERA5 reanalysis dataset, which served as the reference data. ERA5 is the fifth iteration of the atmospheric reanalysis product provided by the European Centre for Medium-Range Weather Forecasts (ECMWF). It offers air temperature images at a reference height of 2 m. The dataset has a spatial resolution of 0.25° × 0.25° (~31 km) and hourly temporal resolution. Some studies have already tested the accuracy of ERA5 [49,50] and concluded that it is sufficient for regional atmospheric studies [51]. The ERA5-Land data for 4 April 2023 were obtained at the same observation time as the Terra MODIS satellite to ensure consistency in the temporal comparison. However, since the spatial resolution of ERA5-Land is lower than the resolution used in this study’s models, there might be some discrepancies in the accuracy when comparing finer-scale temperature patterns. Figure 4 illustrates the spatial distribution of ERA5 mean 2 m air temperature over Korea, focusing on the test bed area used for validation in this study. Despite this limitation, ERA5-Land provides a reliable benchmark for validating the overall NSAT estimation accuracy across the different models in this diverse test bed area. It is worth noting that while recent studies of a similar nature have employed validation techniques, only one other study of NSAT estimation used ERA5 for validation [20], others used meteorological stations [52,53], and some did not use any external validation [19,54,55,56].

## 3. Results and Discussion

### 3.1. Feature Importance

**Figure 5:**
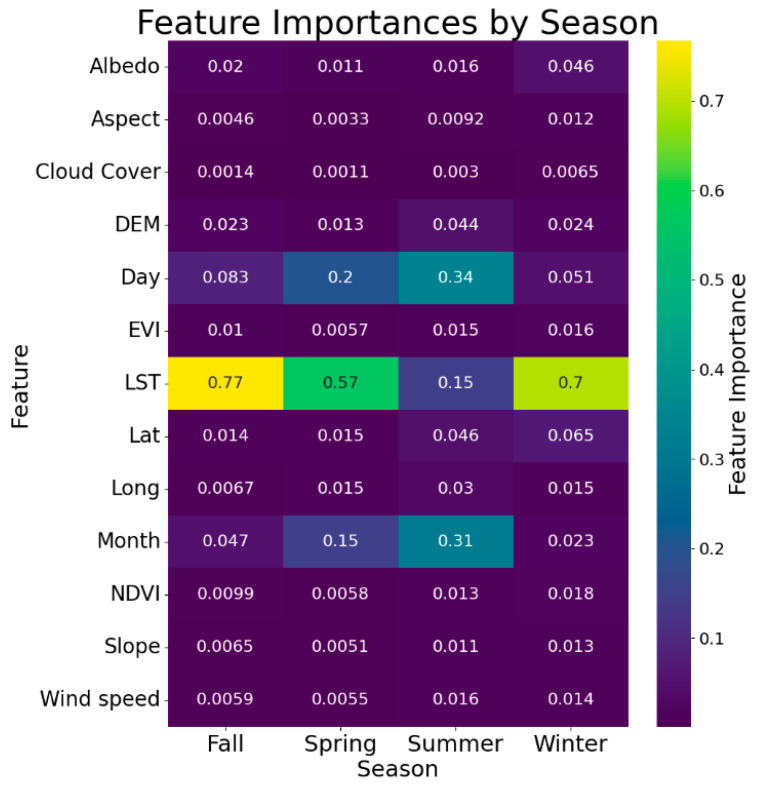
Feature importance of the different X-variables per season.

Shown in Figure 5 is the feature importance of each of the X-variables for the different seasons. The figure shows that the LST is the most critical feature for predicting the air temperature, consistently showing high importance across all the datasets and seasons, a finding that is consistent with other studies [57,58,59]. Temporal features such as the day and month also play significant roles, capturing essential seasonal and daily temperature variations. Several studies demonstrate that incorporating temporal variations like seasonal cycles impacts the model accuracy when predicting temperature patterns in climatic stations [59]. Although not as significant, other variables still have some level of importance to different seasons. The geographical location (latitude and longitude) and DEM have higher importance than other variables in all the seasons, reflecting the importance of the geographical variation and altitude in terms of the temperature distribution [60]. The EVI and NDVI are particularly relevant during summer and winter due to the impact of temperature on vegetation cover or lack thereof [61,62]. During the same seasons, the wind speed becomes more significant as well, reflecting the influence of atmospheric circulation on temperature regulation during these times of the year [63]. On the other hand, while the albedo has higher significance during winter, due to snow cover’s impact on the surface temperature [64,65], the variable still does have some level of importance in the other seasons when compared to the remaining variables.

Given the varied importance of unique features across seasons, feature selection becomes critical in optimizing the performance of the different NSAT models. The next discussion focuses on the results of the feature selection process employed for the ensemble models that employ optimized parameters (SEOP and SEOPPS), and if these key variables align with the results of the observed feature importance.

**Figure 6:**
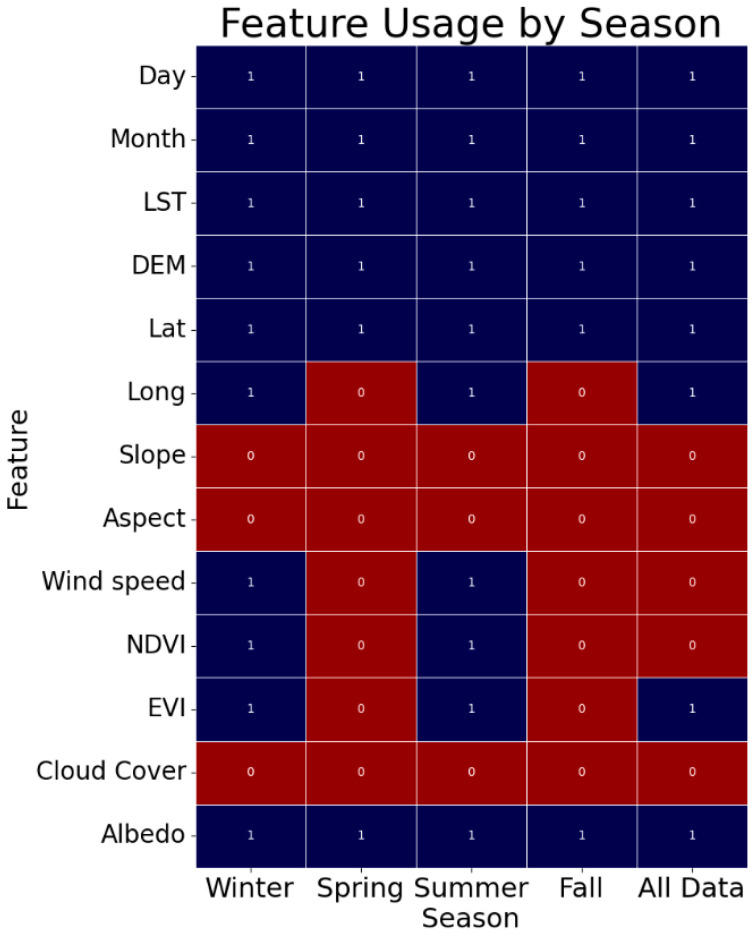
Feature selection for the SEOP and SEOPPS models by season: 1 indicates selected features, while 0 indicates unselected features.

Shown in Figure 6 are the selected variables for the SEOP and SEOPPS models. A designation of “1” signifies features that have been selected, whereas a designation of “0” indicates features that have not been selected. Key features such as the day, month, LST, DEM, latitude, and albedo demonstrate consistent selection across all the seasons and the overall model. The features selected align with the discussion earlier on the feature importance, where the same variables gathered the higher importance values among the unique features across the different seasons. The season-specific feature selection features longitude, NDVI, EVI, and wind speed were selected for the summer and winter seasons. Lastly, the EVI was also selected for the SEOP model. The broader selection of variables during summer and winter reflects the greater variability and complexity of the temperature patterns during extreme weather conditions, where factors such as wind patterns and vegetation cover become more significant [66,67], while the selection of the EVI for the SEOP model underscores that there is still a need for a vegetation index in the overall model.

### 3.2. Model Results and Accuracy

**Figure 7:**
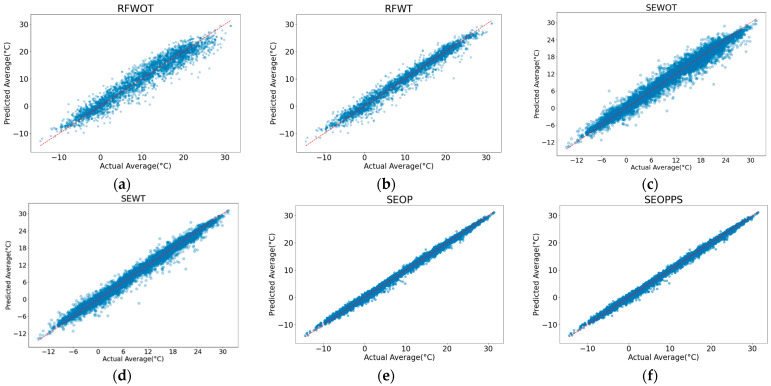
Scatter plots comparing the actual versus predicted average NSAT for the various modeling approaches. The plots illustrate the performance of different models: (**a**) RFWOT, (**b**) RFWT, (**c**) SEWOT, (**d**) SEWT, (**e**) SEOP, and (**f**) SEOPPS. The closer alignment of points to the diagonal line indicates higher prediction accuracy, demonstrating the progressive improvement in model performance with the inclusion of temporal features, ensemble methods, and parameter optimization.

After analyzing the feature importance and selection of the variables, we now analyze the accuracy of the results of each of the models. Table 3 summarizes the performance metrics, while Figure 7 visually represents the models’ performances by displaying the predicted vs actual NSAT. As seen in Table 3, all the models exhibit high R^2^ while having varying degrees of CV R^2^, indicating that while all the models fit the training data well, not all of them can perform well on the validation data. Therefore, the following discussion in this section will focus on the CV R^2^ rather than the R^2^.

Based on the results, we can see that RFWOT, serving as the baseline model, shows the highest RMSE of 2.480 and MAE of 1.906, and a CV R^2^ of 0.765. The high error and low CV R^2^ highlight the limitations of the model, which does not consider temporal features. This is also reflected in the scatter plot in Figure 7, where a significant spread of data points is observed, indicating substantial prediction errors. By incorporating temporal data (day and month), the new model, RFWT, demonstrates an observable improvement over its predecessor. The RMSE was reduced to 1.685 °C and the MAE to 1.240 °C, with an enhanced CV R^2^ of 0.881. The scatter plot for RFWT also exhibits a tighter clustering of points compared to RFWOT. The first of the ensemble models, the SEWOT model, achieves an even lower RMSE of 1.442 °C and MAE of 0.983 °C and higher a CV R^2^ of 0.882. This model is again significantly improved by adding the temporal parameters. The newer model, SEWT, has an RMSE of 0.814 °C and MAE at 0.528 °C and higher a CV R^2^ of 0.886. This marks a significant increase in accuracy as it achieved a sub-degree RMSE. However, the scatter plot figure does reveal that there is not too much change in terms of the overall fit of errors in the data points when compared to the first two models. The last two models, SEOP and SEOPPS, demonstrate the highest accuracy and generalizability. The SEOP model achieves an RMSE of 0.536 °C, an MAE of 0.394 °C, and a CV R^2^ of 0.924. The SEOPPS model refines these results even further, achieving an RMSE of 0.534 °C, an MAE of 0.391 °C, and a CV R^2^ of 0.968. Both the scatter plots of these models show a significant improvement in the predicted vs. actual values, as seen in the tight fit of the data points to the X–Y line. With their accuracy, both SEOP and SEOPPS outperform previous studies that do not incorporate temporal and ensemble models in their estimation, showing the significance of both model improvements when it comes to NSAT estimation.

### 3.3. Model Seasonal Analysis

Table 4 summarizes the performance metrics for the SEWOT, SEWT, SEOP, and SEOPPS models across different seasons. It is important to note that the overall trends observed in the seasonal data align with the general improvements seen in the overall model accuracy. The SEOP and SEOPPS models, which demonstrated the best overall accuracy parameters in the full dataset, remain highly consistent across seasons, showing the lowest RMSEs and MAEs in all cases. While SEWT performs comparably to SEOP and SEOPPS during spring and summer, it proves to be less dependable in other seasons. This shows the dependability of the two latter models across all the seasons.

**Figure 8:**
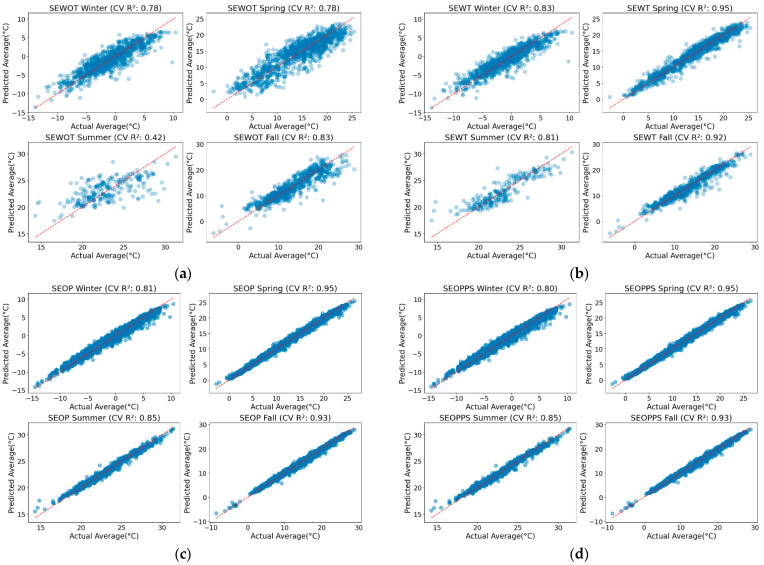
Predicted vs. actual NSAT values for the (**a**) SEWOT, (**b**) SEWT, (**c**) SEOP, and (**d**) SEOPPS models by season: winter (top left), fall (top right), summer (bottom left), and spring (bottom right).

To give a different perspective on the seasonal analysis, Figure 8 is presented. These predicted vs. actual scatter plots help uncover finer details that other performance metrics like the RMSE, MAE, and CV R^2^ might not reveal.

As seen in Figure 8, a consistent pattern emerges in all the models, showing that the inclusion of temporal features and optimized parameters significantly enhances the fit between the predicted and actual values. While the CV R^2^ values for the SEOP, SEOPPS, and SEWT models are close, the scatter reveals differences in the models’ performance. The SEOP and SEOPPS seasonal scatter plots show a much tighter fit compared to SEWT. This suggests that SEOP and SEOPPS not only maintain an elevated level of overall accuracy but also manage to capture the NSAT variations more consistently across different ranges of the data. In contrast, SEWT, while still highly accurate during spring and fall, shows a slightly wider spread of data points in the scatter plots across all the seasons, indicating that the high CV R^2^ alone may not fully capture the finer variations in the prediction accuracy.

Overall, the scatter plots indicate a progressive improvement in model performance from SEWOT to SEOPPS, with the latter models showing more tightly clustered data points and better predictive accuracy across all the seasons than the other two models.

In terms of the seasonal accuracy, the analysis reveals that spring and fall generally show higher predictive accuracy. This is likely due to the relatively stable temperature indices in these seasons, which reduce the impact of extreme events [68,69]. In contrast, summer presents more variability, as higher temperature fluctuations and extreme events make it challenging to maintain the same level of predictive accuracy. Winter also shows slightly lower accuracy across the models, likely due to unique seasonal factors such as the snow cover and high albedo, which can disrupt the typical relationship between the LST and the NSAT [41]. This seasonal analysis highlights how the accuracy is influenced by specific environmental conditions, with spring and fall showing more consistent performance, while summer and winter present additional challenges due to greater variability and unique seasonal factors.

Now, while our results indicate that certain seasons, like spring and fall, still perform slightly better due to the stable temperature indices, the overall accuracy across all the seasons remains high. In the SEOPPS model, the difference between the best-performing season (spring) and the worst-performing season (winter) is only 0.20 °C in the RMSE and 0.13 °C in the MAE, demonstrating remarkable consistency across seasonal transitions. This contrasts with other studies where seasonal differences were more pronounced. For instance, in ref. [19], one study reported a 0.73 °C difference in the RMSE and a 0.54 °C difference in the MAE between spring and winter, while another [53] showed a 0.40 °C RMSE difference but did not report the MAE. These results highlight how our seasonal modeling approach effectively minimizes the seasonal discrepancies, providing reliable and stable NSAT estimations throughout the year.

### 3.4. Test Bed Area Validation and Analysis

**Figure 9:**
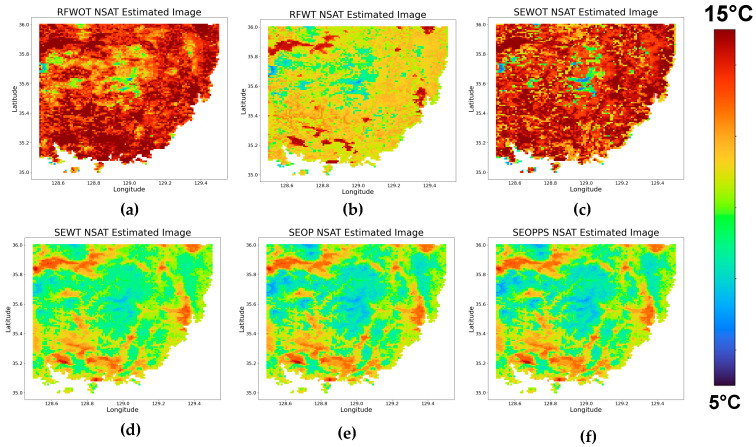
The resulting NSAT prediction images in degree Celsius of the six different models: (**a**) RFWOT, (**b**) RFTW, (**c**) SEWOT, (**d**) SEWT, (**e**) SEOP, and (**f**) SEOPPS.

Having thoroughly analyzed the performance of the various models across different seasons, we now turn our attention to visual assessment of the models, as seen in Figure 9. First, the RFWOT- and SEWOT-estimated NSAT image shows low NSAT variability across different land covers. In the image, there are large patches of higher temperatures, which should only correspond to urban areas but are instead scattered throughout, including areas that should correspond to mountains and vegetation, where lower temperatures would be expected. This suggests that the models struggle to differentiate between urban and natural land cover types. The absence of time-based parameters for both models hindered their ability to capture a more seasonally appropriate temperature. For the RFWT-estimated NSAT image, while it demonstrates a temperature range closer to the other models, it is still significantly higher in terms of the NSAT values compared to the higher-performing models.

The image for the last three models highlights a progression in visual quality compared to the first three models. The SEWT-estimated NSAT image shows a more graduating change in temperature between adjacent areas as well as a better temperature range based on the apparent land cover of the area, i.e., higher temperature in urban areas and lower in vegetation areas. However, when compared to SEOP and SEOPPS, the image produced by SEWT still looks more generalized than the last two models. This is evident due to the smaller temperature range in the vegetation land cover, i.e., less variations of blue. Overall, both the SEOP and SEOPPS models stand out as the most visually coherent. Both models exhibit a finer spatial resolution with more consistent temperature gradients, aligning better with the expected temperature distribution of the land cover. The smoother transitions between adjacent areas and better-defined temperature zones suggest that SEOP and SEOPPS can manage the complexities of downscaling the NSAT more effectively than the other models.

**Figure 10:**
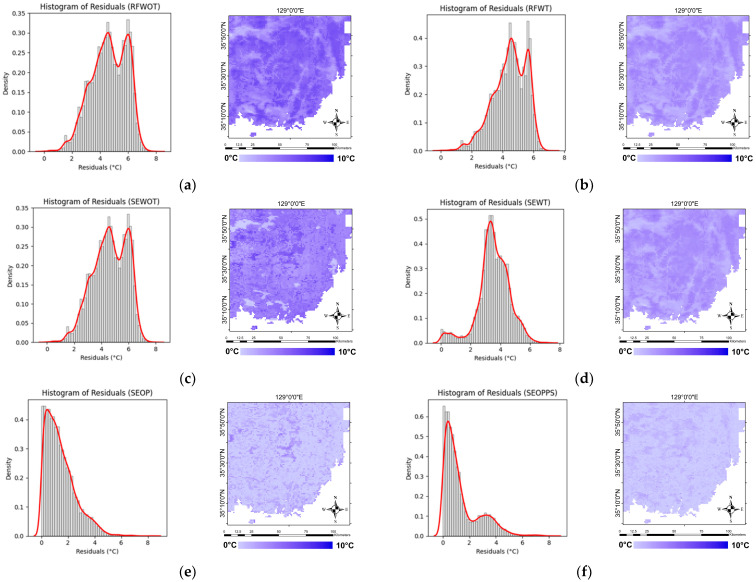
Density plots and map of the absolute residuals for the six modeling approaches: (**a**) RFWOT, (**b**) RFWT, (**c**) SEWOT, (**d**) SEWT, (**e**) SEOP, and (**f**) SEOPPS.

To further assess the models’ performance, we now turn to the analysis of the absolute residuals of the models compared to the ERA5 dataset. The density plots and images of the residuals can be seen in Figure 10. The RFWOT and SEWOT models exhibit a wide spread of residuals, indicating a higher occurrence of larger prediction errors. There is a noticeable improvement with the RFWT and SEWT models, with the residuals clustering more tightly compared to the first two. However, all four models have residuals not around zero, indicating a systematic error, where the predicted values are consistently higher than the reference data. The SEOP and SEOPPS models show the most significant improvement, with the residuals densely concentrated around zero and the narrowest distribution among all the models. This tight clustering, especially in the SEOPPS model, reflects the better performance of these models in minimizing prediction errors. Table 5, showing the MAE and RMSE of the models in the test data, further corroborates this, as the first four models have MAE and RMSE values of ~4 to 5 °C compared to the ~1.5 °C MAE and ~1.9 °C RMSE values of the SEOP and SEOPPS models.

### 3.5. Overall Assessment of the Models

The overall assessment of the models reveals a clear progression in terms of both the accuracy and visual performance as we move from the simpler to more complex models. The RFWOT model had the highest RMSE and MAE values, indicating its inability to accurately estimate the NSAT due to the lack of temporal data. This is reflected visually in its NSAT map, which showed high spatial variability and a lack of alignment with the land cover, suggesting poor downscaling accuracy. The RFWT model, with the inclusion of temporal features, showed a noticeable improvement in the RMSE and MAE values, though the visual output still suffered from lower detail and some artifacts, like the large straight lines, especially in vegetated areas.

The SEWOT model introduced ensemble methods, significantly improving the accuracy over RFWT, with a more coherent visual alignment with the land cover. However, the introduction of temporal features in SEWT further boosted its performance, both in terms of the accuracy and the smoothness of the NSAT estimates. SEWT provided better detail in the visual outputs, reducing the spatial artifacts that were prominent in the previous models. Seasonal analysis also showed that SEWT performed better than SEWOT across all the seasons, particularly in spring and fall, which had the most predictable temperature patterns.

The SEOP and SEOPPS models delivered the highest accuracy, with both models showing almost identical RMSE and MAE values across all the seasons. Visually, both models offered elevated levels of detail and accurate temperature gradients across different land covers. While SEOPPS slightly outperformed SEOP, the difference in accuracy was marginal. Given that SEOPPS is computationally more intensive, the SEOP model may provide a more efficient solution for most practical applications, without a significant loss in accuracy.

### 3.6. Comparison with Other Studies

As discussed earlier, there have been several studies focusing on the use of remote sensing, more specifically MODIS LST, in NSAT estimation. The most common methods include machine learning techniques and statistical methods. Earlier studies focused on monthly or broader temporal scales [43,46], while more recent ones focused on the daily mean [52,53,70] and, just like ours, instantaneous at the satellite pass time. Two of those studies, one in Israel [54] and one in China [19], respectively, compared several machine models and found out that RF performed the best compared to SVM, ANN, multiple linear regression, and ordinary kriging. For the studies that performed instantaneous estimation, the ones with the best accuracy used RF (RMSE = 1.29 °C) [19] and PS-DL (RMSE = 0.89 °C) [20]. Below, Table 6 shows the details of the comparison of our study with other recent studies involving NSAT estimation.

From our study, we observe that aside from the LST, which is used by all the studies, the most common variables are the DEM and NDVI [19,52,54,55,56,70]. Both of these variables were also included in our best-performing model (SEOPPS). However, our second best model (SEOP) used the EVI instead of the NDVI. Only one other study aside from ours incorporated temporal parameters, but it applied them to daily maximum air temperature readings rather than intra-daily instantaneous values [56]. In terms of the number of variables used, the SEOPPS model utilized eight variables, while the other studies were between seven and nine [19,54,55,56] and less than five [20,52,53,70]. Regarding the methods, RF was the most commonly applied, while other studies employed statistical methods, regression analysis, and DL techniques. None of these studies, however, separated their models by season, which limits their ability to capture seasonal variations effectively. Additionally, these studies did not perform a comprehensive feature importance analysis to identify and prioritize the most impactful variables, relying instead on predefined or literature-based feature sets. In terms of the accuracy, our study outperformed those using similar variables and even those employing the same method (RF) [19,54,55] or more advanced approaches [20,56]. Despite using fewer stations and employing a less advanced model than some of the other studies, our approach achieved lower RMSE and MAE values. This suggests that a well-tuned model with optimized feature selection can rival or even exceed the accuracy of more complex methodologies.

### 3.7. Limitations and Future Work

In the current study, certain variables were excluded due to limitations in data availability or their minimal overall influence on the estimation of the NSAT. This study’s scope is further defined by a daily temporal resolution and a focus on the geographic region of Korea. Notably, precipitation data and a distinct snow cover variable were excluded. While both precipitation and snow cover have the potential to exert a significant influence on temperature measurements, these variables were disregarded in light of this study’s parameters and the data at hand.

Precipitation data: The exclusion of continuous spatial precipitation data was necessitated by the inadequate availability of comprehensive, pixel-level datasets. In lieu of this, cloud cover was introduced as an auxiliary variable, thereby indirectly addressing the conditions typically associated with precipitation. Nevertheless, the acquisition of a spatially continuous precipitation dataset would facilitate a more precise characterization of rainy days and their repercussions for the NSAT, marking this as a prospective enhancement for subsequent research endeavors.

Snow Cover: The high albedo of snow cover can influence the NSAT by reflecting a substantial portion of solar radiation, consequently resulting in a reduction in the land surface temperature (LST). To encapsulate some of these effects, the LST was employed as an indirect proxy for snow cover, particularly during the winter months. Although this methodology reflects certain cooling effects linked to snow, it does not comprehensively address the distinct reflectivity characteristics of snow-laden surfaces. Incorporating dedicated snow cover data, such as those derived from MODIS, could further augment the precision of winter-specific models in forthcoming studies.

Furthermore, for the seasonal classification, this study used the conventional seasonal month groupings as defined by the KMA: spring (March–May), summer (June–August), fall (September–November), and winter (December–February). This approach provides a standardized framework across all the stations but may not account for regional variations in seasonal onset that can be set by a more date-specific start of a season.

These constraints underscore opportunities where enhanced data and refined methodologies could bolster the model’s accuracy and versatility, specifically across diverse seasonal and environmental contexts. Addressing these considerations in future inquiries would contribute to a more thorough understanding and improved estimation of the NSAT across varying weather and surface scenarios.

## 4. Conclusions

This study successfully addressed the primary research questions by identifying the key spatio-temporal factors influencing the LST–NSAT relationship, integrating these factors into machine learning models, and determining the best method for handling seasonal data. The analysis showed that the date and location were the most influential spatio-temporal factors, alongside environmental parameters such as the LST, EVI, and DEM, in shaping the LST–NSAT relationship across different seasons. This confirms that temporal features significantly impact the NSAT estimation accuracy.

Incorporating these factors into machine learning models proved highly effective, with the models that included temporal data (e.g., RFWT) consistently outperforming those that did not (e.g., RFWOT), highlighting the importance of temporal integration. The ensemble models (SEWOT and SEWT) further improved the accuracy, demonstrating that combining multiple predictions enhances the model performance across different seasons.

Finally, this study found that the optimized ensemble models (SEOP and SEOPPS) offered the most accurate and consistent NSAT estimates. SEOPPS offered the best performance, but SEOP was close behind. The marginal difference between the performance of the SEOP and SEOPPS models may not justify the extra step in the methodology of the SEOPPS model. In terms of the seasonal analysis, spring and fall were modeled more accurately compared to summer and winter, with winter showing slightly lower accuracy due to specific seasonal factors that could be further explored in future work.

## Figures and Tables

**Table 1:** Data used in this study.

Dataset	Details	Source
NSAT	Daily air temperature	KMA
LST	Daily LST at 1 km	MODIS
DEM	Approximately 90 m	SRTM
Mean Wind Speed	Approximately 300 m	GWA
Albedo	16-day product at 1 km	MODIS
NDVI	16-day product at 500 m	MODIS
EVI	16-day product at 500 m	MODIS
Cloud Cover	16-day product at 1 km	MODIS
Slope	DEM derived at 90 m	SRTM
Aspect	DEM derived at 90 m	SRTM

**Table 2:** Summary and description of the models used.

Model	Model Description
RFWOT	Random forest model without temporal data
RFWT	Random forest model with temporal data
SEWOT	Seasonally informed ensemble model without temporal data
SEWT	Seasonally informed ensemble model with temporal data
SEOP	Seasonally informed ensemble model with optimized parameters
SEOPPS	Seasonally informed ensemble model with optimized parameters per season

**Table 3:** Summary of the accuracy parameters of the different models.

Model	RMSE (°C)	MAE (°C)	R^2^	CV R^2^
RFWOT	2.480	1.906	0.917	0.765
RFWT	1.685	1.240	0.969	0.881
SEWOT	1.442	0.983	0.991	0.882
SEWT	0.814	0.528	0.996	0.886
SEOP	0.536	0.394	0.996	0.924
SEOPPS	0.534	0.391	0.996	0.968

**Table 4:** Performance metrics of the models per season.

Model		RMSE (°C)	MAE (°C)	CV R^2^
SEWOT	Winter	2.460	1.932	0.778
	Spring	2.608	2.110	0.784
	Summer	3.265	2.676	0.491
	Fall	2.230	1.710	0.830
SEWT	Winter	1.568	1.130	0.832
	Spring	1.281	0.982	0.945
	Summer	1.616	1.248	0.805
	Fall	1.336	0.992	0.923
SEOP	Winter	0.631	0.461	0.807
	Spring	0.418	0.315	0.953
	Summer	0.530	0.385	0.850
	Fall	0.469	0.346	0.932
SEOPPS	Winter	0.626	0.447	0.804
	Spring	0.422	0.318	0.952
	Summer	0.536	0.391	0.849
	Fall	0.452	0.341	0.935

**Table 5:** Performance metrics of the models in relation to the test area.

	RFWOT	RFWT	SEWOT	SEWT	SEOP	SEOPPS
MAE	4.629	4.459	4.629	3.552	1.433	1.353
RMSE	4.794	4.587	4.794	3.713	1.902	1.818

**Table 6:** Summary of the comparison with other studies.

Method	Coverage/Number of Stations	Resolution	Variables	NSAT Type	Accuracy			Reference
					RMSE (°C)	MAE (°C)	R^2^	
SEOPPS	South Korea/147	Daily at 1 km	Day, Month, Lat, Long, LST, EVI, DEM, Albedo	Intra-daily instantaneous	0.53	0.39	0.99	This study
RF	Jingjingi, China/1527	Daily at 1 km	LST, Lat, NDVI, DSR, Land Cover, DEM, Declination	Daily mean	1.29	0.94	0.99	[19]
				Daytime instantaneous	1.88	1.35	0.98	
				Nighttime instantaneous	2.47	1.83	0.95	
RF	Region of Murcia, Spain/53	Daily at 1 km	LST, Albedo, NDVI, DEM, Distance to sea, potential isolation, wetness index	Daytime instantaneous	3.01	-	0.89	[55]
RF	Israel/85	Daily at 1 km	LST, NDVI, Road and Population Density, Distance to water bodies, DEM, Slope, Aspect, Urban Fractions, Vegetation Fractions	Intra-daily	1.58	1.12	0.96	[54]
				Instantaneous				
				Daily max	1.89	1.27	0.97	
Statistical Methods	Southeastern USA/538	Daily at 1 km	LST, NDVI, Urban Density, Distance to water bodies	Daily mean	1.38	-	0.97	[70]
GWR	USA/10,141	Daily at 1 km	LST, DEM	Daily min	2.14	1.54	0.95	[52]
Linear Regression	Shaanxi Province, China/23	Daily at 1 km	LST	Daily mean	2.41	1.84	-	[53]
Deep Neural Network	China/829	Daily at 0.01°	LST, NDVI, DEM, Albedo Lat, Long, Land cover, Day, Month, Zenith angle, Road and Population density, wind speed, Soil moisture content	Daily max	2.00	1.54	0.99	[56]
PS-DL	Shandong Province, China/123	Daily at 1 km	LST, Brightness temperature, Emissivity, Water vapor content	Intra-daily instantaneous	0.89	0.78	0.99	[20]

## Data Availability

The data that supports the findings of this study are included in the article. Additional raw data are available from R.J. upon reasonable request.
